# Extensively Drug-Resistant Tuberculosis (XDR-TB) - A Potential Threat in Ireland

**DOI:** 10.2174/1874306400701010007

**Published:** 2007-12-15

**Authors:** Anne Marie Mc Laughlin, Rory A O’Donnell, Noel Gibbons, Mary Scully, Darina O’Flangan, Joseph Keane

**Affiliations:** 1 Department of Respiratory Medicine, CREST Directorate, St James’ Hospital, Dublin 8, Ireland; 2 Irish Mycobacteria Reference Laboratory St James Hospital, Dublin 8, Ireland; 3 Department of Public Health Medicine, Population Division, Health Service Executive, Dr Steevens’ Hospital, Dublin 8, Ireland; 4 Health Protection Surveillance Centre 25-27 Middle Gardiner St Dublin 1, Ireland

## Abstract

We describe a case of a 25 year old female from Lithuania who presented with a productive cough. Chest radiograph demonstrated an infiltrate in the left upper lobe and a cavitating lesion in the right middle lobe. Sensitivity testing of her sputum led to a diagnosis of extensively drug-resistant tuberculosis (XDR-TB). This is the first case in Ireland and highlights the need for physicians to be aware of the possibility of XDR-TB. Moreover it underlines the need for improvement in service provision in terms of a TB reference laboratory and TB clinics.

A 25-year-old female, non-smoker, originally from Lithuania who had been living in Ireland for 2 years presented with a two month history of cough productive of brown sputum. She had no weight loss, night sweats, or dyspnoea. The full blood count was normal, HIV test negative and chest radiograph demonstrated an area of infiltration in the left upper lobe and a cavitating lesion of the right middle lobe (Fig. **1**). Diagnostic investigations confirmed acid fast bacillus in the sputum and she was commenced on anti-tuberculous treatment. Nine days later TB culture demonstrated resistance to all first line agents. She was commenced on oral moxifloxacin, prothionimide, PAS, cycloserine and intramuscular capreomycin. She was discharged home on Directly Observed Therapy (DOT) in close conjunction with our Public Health colleagues.

Five weeks following initial diagnosis, the extensively drug-resistant tuberculosis (XDR-TB) profile (Table **1**), was reported and demonstrated sensitivity to capreomycin, clofazimine, prothionimide and cycloserine. Resistance was noted to amikacin, streptomycin, isoniazid, rifampicin, ethambutol, pyrazinamide, ciprofloxin, clarithromycin, rifabutin and PAS. Second line susceptibility were performed in radiometric Bactec™ in 2 reference laboratories. In view of the resistance, treatment was continued on the 4 drug regimen: moxifloaxin, cycloserine, prothionimide and capreomycin. She became smear negative after 2 months of therapy. After eight months she developed tinnitus and the capreomycin was stopped, audiogram was normal. She received DOT therapy for a total of 20 months. Chest radiograph following 20 months of therapy demonstrated no evidence of active disease or evidence of old fibrocalcific disease.

## DISCUSSION

XDR-TB was first described as an entity in March 2006, in a report jointly published by the US Centers for Disease Control and Prevention (CDC) and WHO [1]. At this point, it was described as a disease caused by strains of *Mycobacterium tuberculosis* that were resistant not only to isoniazid and rifampicin (*i.e.* MDR-TB) but also to at least three of the six classes of second-line anti-TB drugs (aminoglycosides, polypeptides, fluoroquinolones, thioamides, cycloserine and para-aminosalycilic acid). XDR-TB was redefined at a meeting of the WHO XDR-TB Task Force, in October 2006, in Geneva, such that it is now defined as: resistance to at least rifampicin and isoniazid (which is the definition of MDR-TB), in addition to any fluoroquinolone, and to at least one of the three following injectable drugs used in anti-TB treatment: capreomycin, kanamycin and amikacin [2].

Because of its recent description, there is a paucity of published data on the prevalence of XDR-TB. In the USA, 4% of the MDR-TB strains isolated between 1993 and 2004 were XDR-TB. In Europe, representative data are available from Latvia [3] where 19% of the MDR strains isolated from 2000-2002 were XDR and Norway, where an outbreak of 23 cases (15 of them being XDR) has been ongoing for >10 yrs [4]^. ^In Asia, data are available from South Korea [5], where 15% of the MDR-TB strains isolated in 2004 were XDR, and also Iran [6] where 12 (10.9%) of 113 MDR-TB strains isolated were XDR. However it is the emergence of XDR-TB in South Africa that is of particular concern since XDR-TB transmission to HIV co-infected patients is associated with a high mortality rate there [7]. Furthermore, it is estimated that there are >63,000 MDR-TB cases in the countries of the former Soviet Union [8].

The largest European study on XDR-TB has recently been published and it highlights two important points firstly XDR-TB cases are frequently not identified in clinical practice, and of even greater concern that XDR-TB cases have a clinical outcome worse than MDR-TB. This indicates that the XDR diagnosis has important treatment and prognostic values [9]. Attention has also focused on coexistence of HIV and XDR-TB. Of 536 TB patients in the small town of Tugela Ferry in KwaZulu-Natal, 221 were found to have multidrug resistance and of these, 53 were diagnosed with XDR-TB. Fifty-two of these patients died, most within 25 days. Of the 53 patients, 44 had been tested for HIV and all 44 were found to be HIV-positive. The patients were receiving antiretrovirals and responding well to HIV-related treatment, but they died of XDR-TB [10].

Correct and complete treatment of MDR-TB and XDR-TB is vital both for the individual patient and the community. There are no published guidelines on the management of XDR-TB. Guidelines on the management of MDR-TB suggest use of 4-6 drugs [11,12] although the optimal number of drugs used in a retreatment regimen for MDR-TB has never been studied in a clinical trial. As part of the regimen, a daily injectable medication should be used until at least 6 months of negative cultures have been documented and a fluoroquinolone should be used where possible. Additionally DOT, aggressive management of adverse effects, rigorous drug susceptibility testing and adjuvant resective surgery where necessary should be considered. In some cases empiric MDR-TB treatment may be required whilst awaiting results of antimicrobial-sensitivity. In these cases, it is important to commence at least six agents to which the infecting strain is likely to be sensitive, avoiding drugs which the patient has received in the past and bearing in mind local resistance patterns.

The emergence of MDR and XDR-TB in Ireland requires us to take several measures: These include offering TB disease and Latent TB infection (LTBI) screening, followed by treatment when appropriate, to all new entrants from high TB endemic countries. Additionally, we should improve our laboratory capacity for susceptibility testing of second line drugs. We can also improve our Public Health service by ensuring more rapid contact tracing and medication compliance, though easy access to DOT. There is now a responsibility on medical and scientific TB personnel to develop methods to rapidly diagnose smear-negative TB cases, to evaluate new markers of TB infection and disease, to develop new drugs for the treatment of TB and to develop international standards and guidelines for the management of drug resistant TB.

This case highlights an emerging health problem in Europe and underlines the necessity for physicians to be aware of MDR and XDR-TB in patients and most particularly in patients presenting from areas of high prevalence.

## Figures and Tables

**Fig. (1):**
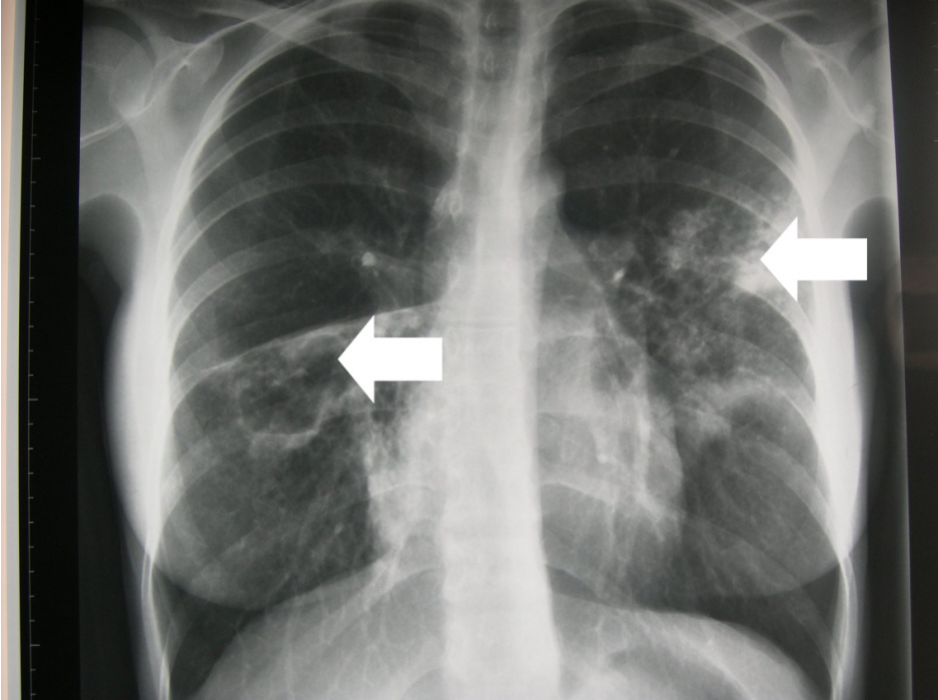
Chest radiograph demonstrating an area of infiltration in the left upper lobe and a cavitating lesion in the right middle lobe.

**Table 1.** Sensitivities of the Isolated *Mycobacterium Tuberculosis*

Antibiotic	Sensitivity/Resistance
Isoniazid	R
Rifampicin	R
Ethambutol	R
Pyrazinamide	R
Streptomycin	R
Amikacin	R
Ciprofloxacin	R
Clarithromycin	R
Rifabutin	R
PAS	R
Capreomycin	S
Prothionimide	S
Clofazimine	S
Cycloserine	S
